# Expressed information needs of patients with osteoporosis and/or fragility fractures: a systematic review

**DOI:** 10.1007/s11657-018-0470-4

**Published:** 2018-05-08

**Authors:** Grace Raybould, Opeyemi Babatunde, Amy L. Evans, Joanne L. Jordan, Zoe Paskins

**Affiliations:** 1 Arthritis Research UK Primary Care Centre, Research Institute for Primary Care & Health Science, Keele University, Keele, Staffordshire ST5 5BG UK; 2 Haywood Academic Rheumatology Centre, Staffordshire and Stoke-on-Trent Partnership Trust, Stoke-on-Trent, ST6 7AG UK

**Keywords:** Information needs, Osteoporosis, Education, Communication

## Abstract

**Summary:**

This systematic review identified patients have unmet information needs about the nature of osteoporosis, medication, self-management and follow-up. Clinician knowledge and attitudes appear to be of key importance in determining whether these needs are met. Unmet information needs appear to have psychosocial consequences and result in poor treatment adherence.

**Purpose:**

Patient education is an integral component of the management of osteoporosis, yet patients are dissatisfied with the information they receive and see this as an area of research priority. This study aimed to describe and summarise the specific expressed information needs of patients in previously published qualitative research.

**Methods:**

Using terms relating to osteoporosis, fragility fracture and information needs, seven databases were searched. Articles were screened using predefined inclusion and exclusion criteria. Full-text articles selected for inclusion underwent data extraction and quality appraisal. Findings were drawn together using narrative synthesis.

**Results:**

The search identified 11,024 articles. Sixteen empirical studies were included in the review. Thematic analysis revealed three overarching themes relating to specific information needs, factors influencing whether information needs are met and the impact of unmet information needs. Specific information needs identified included the following: the nature of osteoporosis/fracture risk; medication; self-management and understanding the role of dual energy x-ray absorptiometry and follow-up. Perceived physician knowledge and attitudes, and the attitudes, beliefs and behaviours of patients were important factors in influencing whether information needs were met, in addition to contextual factors and the format of educational resources. Failure to elicit and address information needs appears to be associated with poor treatment adherence, deterioration of the doctor-patient relationship and important psychosocial consequences.

**Conclusion:**

This is the first study to describe the information needs of patients with osteoporosis and fracture, the impact of this information gap and possible solutions. Further research is needed to co-design and evaluate educational interventions with patients.

**Electronic supplementary material:**

The online version of this article (10.1007/s11657-018-0470-4) contains supplementary material, which is available to authorized users.

## Introduction

UK government policy places emphasis on providing patients with good quality health information, in order to encourage patient participation in healthcare and ensure that patients have greater power, protection and choice in key aspects of their care [1]. As well as promoting patient-centred care, this policy is an important driver in the management of health resources, achieved through emphasis of the importance of self-management. Especially in the context of an ageing population, and with increasing prevalence of long-term conditions such as osteoporosis, a strategy is needed whereby patients accept more responsibility for managing their own conditions which in turn will reduce or thwart the increase in demand on healthcare resources.

Patient information is a key component of effective self-management [2] and specifically in relation to osteoporosis and fracture prevention, information and education interventions have been shown to improve outcomes including health-directed behaviours and positive and active engagement in life, skill and technique acquisition, and social integration and support [3]. Patient education centres on the assumption that patients who are better informed about their condition and management will be more likely to adopt positive health behaviours [4] and will therefore improve, maintain or slow deterioration of their health status [5]. However, this viewpoint of patient education does not acknowledge the role of patient opinions and choice and implies that health professionals set the education agenda and define optimal health behaviours [6].

Patients are often dissatisfied with the information they receive from health professionals. A recent national survey of 1088 supporters of the National Osteoporosis Society (NOS) rated ‘easy access to information from health professionals’ as the number one research priority for osteoporosis and fracture out of 40 domains [7]. The focus groups that preceded this survey emphasised the importance, yet the relative lack, of information given by healthcare professionals early on in the participant’s pathway, e.g. at time of diagnosis, and in ongoing consultations with primary care clinicians [8].

To date, studies that have attempted educational needs assessment in osteoporosis have done so by measuring patient knowledge and inferring educational unmet need based on inaccurate answers to factual surveys [9–11]. These surveys tell us nothing about what patients want to know. Furthermore, quantitative methods fail to capture the context which underlies the reported needs of patients; qualitative research methods facilitate the in depth understanding of the thoughts and perceptions that underlie and influence information needs of patients, facilitating informed approaches to target unmet need [7]. The aim of this study was therefore to describe and summarise *patient expressed* information needs in previously published qualitative research, to better understand the research agenda relating to information needs of patients with osteoporosis and/or fractures [7].

## Methods

### Literature search

The review was conducted based on a pre-established protocol (detailed eligibility criteria are presented in Table 1, and search terms and search strategy in supplementary material 1). A systematic search for qualitative studies on expressed information needs of patients with osteoporosis/fragility fracture was conducted in seven databases: Medline, EMBASE, PsychINFO, Web of science, CINAHL, HMIC and AMED from the start of each database to July 2016. De-duplication of citations and title screening were completed by JJ and GR. Screening of abstracts against eligibility criteria and subsequent full-text reviews were independently completed by pairs of reviewers (GR and either ZP or OB). Grey literature was sought by hand searching reference lists of included studies and those that satisfied most, but not all of the review’s eligibility criteria. Citation tracking of included studies was also conducted in Google Scholar. Disagreements regarding study eligibility were resolved via discussion until consensus is achieved by the team.

**Table 1:** Inclusion and exclusion criteria

Inclusion criteria	Exclusion criteria
- Studies reporting patient expressed information needs or uncertainties - Over half included participants have either osteoporosis and/or a fragility fracture - Participants are adults - Studies are qualitative	- Studies not in English language and for which translations could not be obtained. - Conference abstracts, letters, studies with no empirical results or no full text available. - Information needs of health professionals, carers or family members rather than participants OR those at risk of osteoporosis. - Participants with fractures which are unlikely to be fragility fractures (e.g. major trauma, age majority participants < 50 years). - Articles describing the evaluation of interventions aimed at improving information support without explicit assessment of participant perceived information needs. - Articles describing assessment of participant knowledge without explicit assessment of participant perceived information needs.

### Quality assessment

Eligible studies were independently appraised by pairs of reviewers (GR, AE and OB). Two tools were combined to support this (Supplementary Material 2). Firstly, the Hawker tool [12] was used to assess quality of the included studies as it can be used to evaluate qualitative research studies using different approaches. This tool includes nine domains (title/abstract; introduction/aims; methods/data; sampling; analysis; ethics/bias; results; transferability; usefulness). However, the Critical Appraisal Skills Programme tool for qualitative research was also used to further inform the appraisal of each article as this tool provided more detail about specific methodological issues relating to qualitative research [13]. For each study, individual items on the Hawker tool were judged as good, fair, poor or very poor. Variations in judgements regarding methodological appraisal of the quality of each studies were resolved through discussion with a third reviewer (ZP). No paper was excluded based on quality scores; it has been argued that excluding studies on the basis of quality criteria may exclude insightful studies [14]. However, study quality was considered during the synthesis stage to ensure themes were primarily based on the good or fair quality studies.

### Data extraction

Using a customised data extraction form, information relating to author, title, date, country of origin, research question, method, sample and setting, year of publication, author’s findings and conclusions, from each study, was extracted. Extracted data was checked for consistency and accuracy.

### Synthesis

Extracted data was tabulated and a thematic analysis was conducted. Findings relevant to our review objectives were grouped under the following headings:

- Expressed information needs, i.e. where patient participant(s) was/were described as wanting to know more
- Expressions of uncertainty, i.e. where patient participant(s) was/were described as being confused or unsure
- Findings describing examples where information was given and was helpful
- *Inferred* information needs (i.e. where authors deduce a need based on tested or assumed knowledge rather than patients expressing a need directly)
- Anything else of relevance to giving and receiving of information

Inferred information needs were not included in subsequent stages of the analysis as it did not relate to our research question. We conducted thematic analysis on the data extracted, manually. The first stage was initially descriptive—to identify a taxonomy or classification of the types of information needs identified. Study quality was considered within each theme to ensure themes were not only informed by poor or very poor quality studies. Subsequently, a more interpretative level of coding was applied to explore and determine explanations for initial findings. Next, we revisited the original study findings to ensure our themes represented all the relevant findings. A higher level of abstraction was achieved by grouping subthemes into overarching themes, following an iterative mapping process and team discussions (ZP, GR, OB), through which a conceptual framework was developed.

## Results

**Fig. 1:**
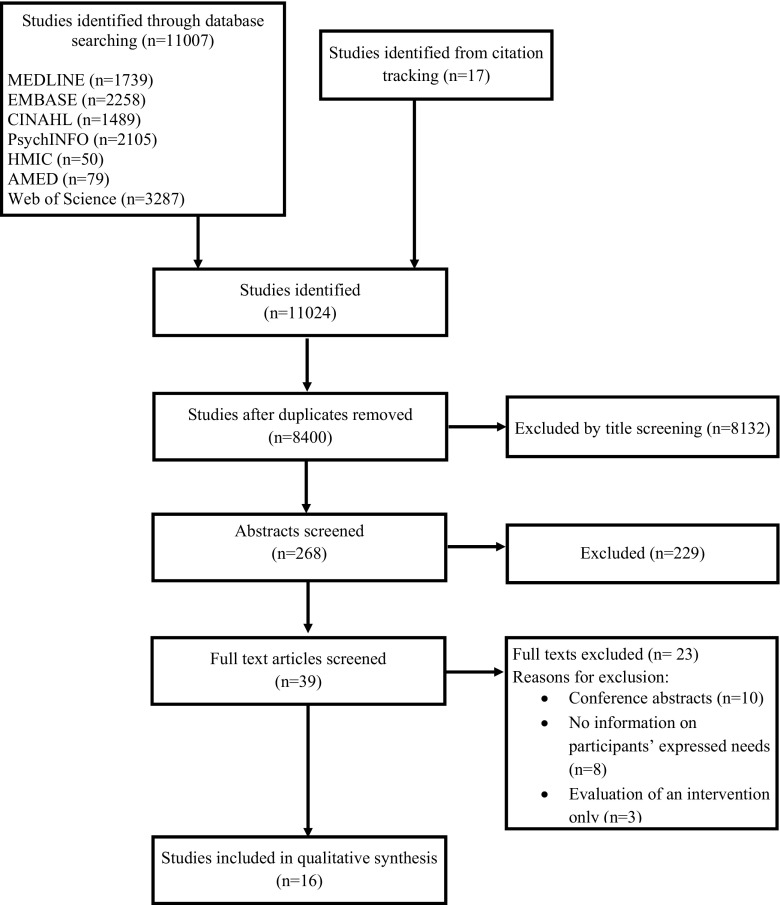
Identification and selection of studies

Eleven thousand seven unique citations were identified by the initial search and a further 17 by reference and citation checking. The review process and study flow is presented in Fig. 1. Sixteen studies were included for data extraction and quality appraisal.

## Summary of included studies

The characteristics of the included studies are summarised in Table 2, including research question, country of origin and methods. Eleven studies utilised single semi-structured interviews [15, 17, 20–23, 25, 27–30] and focus groups were used as method in the remainder [16, 18, 19, 24, 26]. Eleven of the 16 included studies recruited patients from secondary care populations [15–18, 22–26, 29, 30], with the rest recruiting from primary care [20, 27], community or mixed settings [19, 21, 28]. Three studies included patients with specific fracture types (two hip and one vertebral) [22, 28, 30], and in these the research question and information needs related to the specific fracture care in addition to general issues pertaining to osteoporosis [22, 28, 30]. Seven studies were primarily concerned with attitudes and perceptions regarding medication or supplements [15–20, 27], with the others focused on experience of living with the condition more generally. Only three studies had research questions that were directly related to information needs [23, 25, 28]. One study aimed to explore knowledge after an education programme; although this was not directly relevant to our research question, the qualitative approach used did elicit directly *expressed* information needs [26]. For the remainder studies, only a small proportion of the total findings relating to information needs were relevant to our research question and extracted.

**Table 2:** Characteristics of included studies

First author (year), reference	Aim	Method	Sample	Recruitment setting	Country of origin
Besser (2012), [15]	To explore how patients with osteoporosis perceive their illness and treatment in order to investigate adherence and how to improve it.	Semi-structured interviews	*n* = 14 10 with osteoporosis, 4 with osteopenia. All female, mean age 69.	Secondary care—osteoporosis screening unit and rheumatology clinic.	UK
French (2005), [16]	To identify barriers to following calcium recommendations in women with a reduced bone mineral density.	Focus groups	*n* = 30 13 diagnosed osteopenia, 17 diagnosed osteoporosis. All female, age 52–87 years.	Previous osteoporosis treatment programme at a tertiary referral centre. Participants previously had a consultation with a dietician.	Canada
Hansen (2014), [17]	To investigate women’s experiences of living with osteoporosis during the first 6 months after diagnosis when treatment was first prescribed.	Semi-structured interviews	*n* = 15 All with recently diagnosed osteoporosis. All female, age 65–79 years.	Secondary care, following recently attending a DXA scan, at 1 of 2 hospitals.	Denmark
Iversen (2011), [18]	To determine factors influencing adherence to osteoporosis medications among older adults.	Focus groups	*n* = 32 All diagnosed osteoporosis. 30 female, 2 male, age 65–85 years.	Advertisements in a tertiary hospital medical centre newsletter.	USA
Lau (2008), [19]	To explore the experiences and perceptions of postmenopausal women regarding strategies to improve adherence to osteoporosis therapy.	Focus groups	*n* = 37 All with diagnosed osteoporosis and either 1 prescription medication or over the counter medication for osteoporosis. All female, age 48–88 years.	By 3 family physicians, 1 geriatrician, 1 rheumatologist, and 3 community pharmacists.	Canada
Mazor (2010), [20]	To explore older women’s views about prescription osteoporosis medication use and identify factors that influence these views.	Telephone interview	*n* = 30 All diagnosed osteoporosis. All female, mean age 73.4 years.	Primary care.	USA
McKenna (2008), [21]	To compare experience of osteoporosis in Caucasian and South Asian women during consultations.	Semi-structured interviews	*n* = 21 All with osteoporosis, 19 with history of fragility fractures. All female, age 43–82 years.	Support groups, osteoporosis exercise classes, South Asian community centres.	USA
McMillan (2014), [22]	To explore post discharge concerns of older people after a fall-induced hip fracture.	Semi-structured interviews	*n* = 19 All with previous hip fracture. 15 women, 4 men. Age 67–89 years (mean 79).	Secondary care following recent discharge from hospital following hip fracture.	UK
Meadows (2005), [23]	To explore women’s post fracture experiences to understand how general practitioners could tailor patient education about risk.	Semi-structured interviews	*n* = 22 All with previous fragility fracture. All women, over 40 years of age.	Orthopaedic trauma surgery database.	Canada
Nielsen (2011), [24]	To understand how men experience having osteoporosis and handle osteoporosis in their everyday lives.	Focus groups	*n* = 16 All with osteoporosis. All male, age 51–82 years.	Attendees at endocrinology clinic.	Denmark
Nielsen (2010), [25]	To understand the importance of information and knowledge about osteoporosis for participants’ way of handling osteoporosis in their everyday lives.	Semi-structured interviews	*n* = 26 All with osteoporosis. 20 women, 6 men, age 50–84 years. 14 from UK, 12 from Denmark.	By healthcare professionals in a secondary healthcare environment.	UK, Denmark
Sale (2010), [26]	To examine fracture participant understanding of osteoporosis and osteoporosis care after being screened for, and educated about osteoporosis, in a fracture clinic.	Focus groups	*n* = 24 All with a previous fragility fracture. 18 women, 6 men, age 47–80 years.	Urban osteoporosis screening fracture clinic.	Canada
Salter (2014), [27]	To describe key perceptions that influence older women’s adherence and persistence with prescribed medication when identified to be at a higher than average risk of fracture.	Semi-structured interview	*n* = 30 Above average risk of subsequent fracture and recently started preventative osteoporosis medication. All women, age 73–85 years.	Participants of a previous primary care-based randomised controlled trial (SCOOP).	UK
Schiller (2015), [28]	To understand the recovery phase after hip fracture from the patient perspective, and identify specific messages that could be integrated into future educational material for clinical practice to support patients during recovery	Semi-structured interview	*n* = 19 11 with previous hip fracture (10 women, 1 man), 8 family members or other caregivers.	Newspaper advertisement, emails, previous study participants, community organisations with a history of collaboration, hospital research buildings and websites.	Canada
Solimeo (2011), [29]	To explore the experiences of male osteoporosis patients	Semi-structured interview	*n* = 23 13 with previous fragility fracture, 17 on osteoporosis medication. All men, age 53–86 years.	By physician in secondary care.	USA
Svensson (2016), [30]	To understand the lived experience of women with an osteoporotic vertebral compression fracture	Semi-structured interview	*n* = 10 All with vertebral fracture. All women, aged > 65 years.	Secondary care in an outpatient clinic.	Sweden

## Summary of findings relating to quality appraisal

The findings related to quality appraisal are summarised in Table 3. Individual scores for quality appraisal by authors GR, AE and OB were congruent in 115/144 (79.9%) domains. Nine studies were scored as ‘fair’ or ‘good’ in all domains [15, 17, 19–22, 24, 25, 27]. Studies scoring ‘poor’ or ‘very poor’ did so in domains relating to sampling, ethics and bias, analysis, results, transferability and implications. The most common limitation of the included studies was failure to adequately describe the characteristics of the participants. For example, age was discussed regarding eligibility criteria, but the ages of included participants were not included [26, 28]. Limitations of the sampling or recruitment also affected the transferability and implications of the findings [26].

**Table 3:** Quality appraisal of included studies

First author (year)	Abstract, title	Introduction, aims	Method, data	Sampling	Data analysis	Ethics, bias	Results	Transferability	Implications	Comments
Besser (2012) [15]	4	4	4	3	3	3	4	3	3	Single centre. Purposive sampling. Analysis conducted by > 1 author.
French (2005) [16]	3	3	4	2	3	3	4	2	4	Sampling from 1 centre where patients saw a dietician.
Hansen (2014) [17]	3	4	4	3	4	4	4	3	3	Recruitment from 2 sites. Analysis described in depth and conducted by > 1 author. Topic guide not presented, little discussion of limitations beyond author’s role.
Iversen (2011) [18]	4	4	4	3	3	2	4	3	3	Implied consent.
Lau (2008) [19]	3	4	3	3	4	4	4	3	3	Single centre. Purposive sampling. Author demonstrates reflexivity. Minimal description of coding.
Mazor (2010) [20]	4	4	4	4	4	3	3	3	3	Single centre study. Purposive sample. Analysis and topic guide clearly described
McKenna (2008) [21]	3	4	3	3	3	3	4	3	3	Multiple recruitment strategies from one area. Analysis conducted by > 1 author. No discussion of limitations
McMillan (2014) [22]	4	4	4	3	4	3	3	3	4	Purposive sampling. Recruited from 3 sites
Meadows (2005) [23]	4	2	3	2	3	3	3	2	3	Aim not clearly stated. Convenience sample.
Nielsen (2011) [24]	3	4	4	3	4	4	3	3	4	Men recruited from single site. Analysis described in depth. Author demonstrates reflexivity.
Nielsen (2010) [25]	3	4	4	3	3	3	4	3	3	Multi-site study. Analysis and topic guide described in depth. Author demonstrates reflexivity
Sale (2010) [26]	4	4	4	3	3	4	3	2	3	Participants from a standardised programme in 1 fracture clinic.
Salter (2014) [27]	4	3	4	4	3	3	4	4	4	Longitudinal qualitative study embedded within a multi-centre trial. Purposive sample. 2 interviews per participant. Topic guide and analysis clearly described
Schiller (2015) [28]	3	4	3	4	3	3	4	3	2	No clear message for practice or further research
Solimeo (2011) [29]	3	2	3	3	3	3	3	3	2	Lacking implications beyond need for gender-sensitive treatment protocols
Svensson (2016) [30]	4	4	4	3	4	3	2	3	3	No quotes to support findings. No attention to deviant cases.

Scores represent quality ratings: 1 = very poor, 2 = poor, 3 = fair, 4 = good

## Main findings

**Fig. 2:**
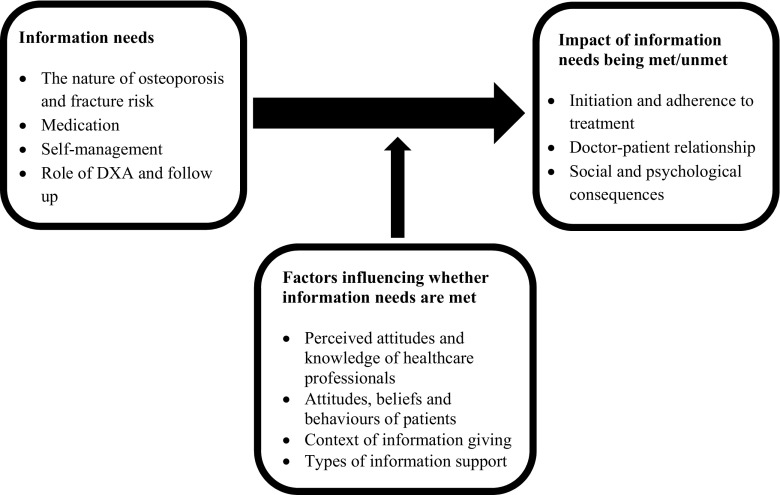
Nature and consequences of unmet educational needs and factors promoting and impeding information being given and received

The main findings are summarised in Table 4. Of the 16 included studies, 13 discussed directly expressed information needs and 12 studies discussed uncertainties. Thematic analysis of the findings revealed three overarching themes discussed individually below, and displayed in the conceptual framework in Fig. 2.

**Table 4:** Authors reported main findings and extracted information needs

	Authors’ main findings	Expressed needs relating to information	Expressed uncertainties	Other findings regarding information needs
Besser (2012) [15]	Patients are unaware that medications reduce fracture risk. Drawings can be used to help patients understand the condition. Some have limited knowledge about causes. Uncertainty about whether osteoporosis can be controlled with medication. Patients who do not attend clinics are particularly at risk of non-adherence.	Feedback on DXA scans. Information about medications (directions, indications, long-term effects). More information about osteoporosis generally.	Cause of their osteoporosis. Role of medication in treating osteoporosis. Seriousness of their condition. Role of diet and self-management in treating osteoporosis. Medication instructions	Current information not perceived as understandable. Perceived negative relationships with their doctor and perceived lack of clinician knowledge were associated with information needs being unmet. Information sought from elsewhere, e.g. family. Use of diagrams to aid explanation during consultations considered helpful. Feedback on DXA scan facilitated compliance.
French (2005) [16]	Health concerns, lifestyle, food preferences and side effects need to be considered in individualised assessments.		The best foods to eat. The best supplements to take.	Inconsistent dietary advice. Not all information sought from health professionals.
Hansen (2014) [17]	Women accept the diagnosis of osteoporosis in different ways, influenced by positive or negative experiences of the diagnosis process. Need for improved support for women to gain understanding of their diagnosis, fracture risk and learning to live with osteoporosis.	Harm of medication.	Whether they had weighed up information appropriately to make decision about treatment.	Having information needs met contributed to a feeling of being taken seriously. One participant tried to give her GP information but it was rejected. Uncertainties associated with a cycle of worry and fear, exacerbated by not getting information from GP.
Iversen (2011) [18]	Patients report lack of knowledge, dissatisfaction with doctor visits, side effects and difficulty complying with or remembering medication instructions as barriers to adherence.		Purpose of medication. How to take medication.	Not enough time in consultations to raise medication issues. Wanted and valued written information. Participant reported primary care physicians needed more knowledge about medications (compared to specialist).
Lau (2008) [19]	Strategies that facilitate adherence to bisphosphonates include having a system to take medication, using cues, being well informed about the reasons for medication and having regular follow-up for support and monitoring.	More information on expected effects of medications and instructions on how to take. Follow up after medication. Suggestions for managing medications more easily. Understandable and written information about medication.	Conflicting messages about medication were given by different healthcare providers.	Participants take in a small proportion of the information given by a specialist. Pharmacists perceived to have more time than physicians to give medicine information. Physicians overly focus on medication. Active in seeking information on medications, from a variety of sources.
Mazor (2010) [20]	Women need clear information about their condition including the diagnosis implications, treatment options and side effects.	Unanswered questions relating to reasons for procedures for taking bisphosphonates and the ‘pros’ of treatment.	Need for drugs if already following lifestyle measures. Need for medication and whether it was safe and effective.	Did not always voice concerns with physician. Difficulty forming questions to ask physician described, and a feeling the doctor would not have time to answer questions. Doctors ‘too quick’ to recommend medication.
McKenna (2008) [21]	In general, patients differed in their views by age more than by ethnicity. South Asian and older participants expressed preferences for receiving information from their GP. Physical activity was inconsistently recommended by their physician.	More information regarding self-management from their doctors including recommendations for exercise.	The role and interpretation of a DXA scan.	Preference for more information through ongoing discussion in sequences of consultations. Lack of confidence to ask questions. Lack of confidence in GPs’ understanding. Inappropriate focus on medication rather than self-management in consultations. Younger participants expressed information needs more than older participants. Ethnicity associated with consultation behaviour, sources of information accessed and readiness to accept information from third parties, e.g. NOS. Participant described how her GP had become more interested in osteoporosis and learned from her, over time.
McMillan (2014) [22]	Participants require information to ‘balance risk’ of physical activity against risk of fracture.	Feedback on their progress after hip fracture.		Valued explanation by physiotherapists about which exercises to do, the purpose of exercises. Missed opportunities to receive information in hospital due to various contextual factors. Repeated consultations with written and verbal information enhanced understanding. Involving relatives in providing information was important.
Meadows (2005) [23]	Attitudes of women with prior low trauma fractures in mid-life toward further fracture risk fell into 3 groups: laissez faire (prefer to wait and see); those who thought they should be doing more, those who were proactive in seeking information and addressing risk.		Supplement use after fracture had healed.	Expressed uncertainties did not lead to taking physician’s advice. Sought information from a variety of sources. Some gain information ‘passively’.
Nielsen (2011) [24]	Maintaining physical activity and maintaining a masculine identity was important to participants. Osteoporosis was seen as a female condition and therefore patient information was perceived as not relevant.	Support groups. Opportunities to talk to other male osteoporosis patients.		Majority did not express information needs. Not asking questions or seeking care due to fear of the future or fear of consequences for employment. Decision to seek care often driven by female partners. Osteoporosis was seen as a female condition; shame and embarrassment.
Nielsen (2010) [25]	Previous life conditions influenced the way osteoporosis was handled. Some patients dealt well with the risk of fracture and pain whilst others were more fearful and limited. Patient information and a good relationship with health professionals were highly valued.	More readily available contact with healthcare professionals. Opportunities to learn from other patient experiences.	Role of medication. Relationship between osteoporosis and normal ageing bone. Prognosis and outlook. Safety of carrying out activities of daily living.	Variation in the amount of information wanted. Some expressed no information needs or that information could be detrimental, e.g. becoming depressed after meeting someone with severe osteoporosis.
Sale (2010) [30]	Despite participants having partaken in a standardised screening programme, in which education was thought to be implicit, ‘ambiguity’^a^ around diagnosis, testing and treatment were described.	Self-management options available alongside medication (dietary sources of calcium/vitamin D). Purpose of bone density scans, why only some body regions are scanned.	Relationship of pain to osteoporosis. Role of supplements, optimum doses. Purpose of medication.	Some participants described discontinuing medication when they ‘felt better’.
Salter (2014) [23]	Adherence to preventive medication for osteoporosis is complex and multifaceted. Individual understanding, choice, risk and perceived need interact; unpredictable patterns of usage and acceptability. Professionals should not assume adherence.	More regular reviews to see if medication was still necessary, and if medication was working.	Whether medication was working. Nature, importance of fracture risk Relationship between falls and fractures; some think medication would reduce falls, or high fracture risk means being a faller.	Some perceived their fracture risk was normal for age and therefore questioned the need to take medication. Long-term change of osteoporotic bones was a lower priority than other illnesses, for participants and their doctors.
Schiller (2015) [25]	Participants discussed 3 methods that enabled recovery: seek support, move more, preserve perspective.	Community resources available to help post-diagnosis. Information focusing on the timeline of their prognosis—a ‘recovery map’ or checklist suggested. Valued information about mobilisation, exercise programmes.		Recognition that asking questions of health professionals can be challenging for some individuals. A patient advocate was suggested as a possible solution by a relative.
Solimeo (2011) [27]	Men feel protected from osteoporosis (‘a female condition’) which can delay diagnosis. Perceived lack of treatment options for men. Limited activities after diagnosis impact on masculine identities.	Medication that is suitable for men (expressed as an unknown, rather than an information need).		Difficulty getting a diagnosis and having to ‘persuade’ their doctor for tests. Need for more ‘marketing’ about osteoporosis affecting men was expressed.
Svensson (2016) [28]	Most prominent experiences of participants with vertebral fracture were fear and concern. 5 themes identified: struggling to understand a deceiving body, breakthrough pain fuelling fear, fearing a trajectory into isolation, concerns of dependency, fearing an uncertain future.	Information on managing vertebral fractures, pain relief and advice to rest. Support to live independently without fear	Uncertain future which contributed to fear	Not taken seriously by healthcare professionals, felt isolated. Felt health professionals thought they ought to accept the changes to their body as ‘normal ageing’. Healthcare professionals were described as uninterested and indifferent to their condition, viewed as low priority. Difficulty getting vertebral fracture diagnosis; participants repeatedly requesting x-rays or referral

^a^The authors used frequent descriptions of ‘ambiguity’ to mean the information could be interpreted in multiple ways thus giving rise to doubt or uncertainty. As this was the authors conjecture, we only extracted information relating to uncertainty where it was clear participants had directly expressed uncertainty

### Specific information needs

Information needs were illustrated in all of the studies. The needs related to some or all of the following: the nature of osteoporosis and fracture risk; medication; self-management and/or the role of dual-energy X-ray absorptiometry (DXA) and follow up.

### The nature of osteoporosis and fracture risk

The relationship between osteoporosis and age was a topic of uncertainty, causing some to question the need for medication for a condition that maybe considered normal for age [25, 27]. Doubt was expressed that osteoporosis was asymptomatic with participants in one study attributing a range of symptoms to the condition including pain, rotting teeth and flaking nails [15]. Findings from a further focus group study suggested that participants felt musculoskeletal pain experienced on activity was a signal of an imminent fracture [26]. Participants sampled from a study of screening for osteoporosis described uncertainty regarding communication of fracture risk, leading some to question the validity of the assessment [27]. The prognosis of osteoporosis, and the seriousness of osteoporosis, was a cause of uncertainty, which resulted in fear of the future [15, 30]. In a similar vein, patients recovering from hip fracture wanted to know in more detail what recovery would like and what the key ‘milestones’ would be [28].

### Medication

Uncertainty about the purpose of medication was described in several studies [15, 18–20] and half of the participants in the interviews reported in a study published in 2012 had not considered that medication would reduce fracture risk [15]. In one study, a participant described a perception that bisphosphonates would reduce risk of falls [27]. One study focused on views on medication, participants described wanting to know more about the ‘pros’ of treatment [20]. Information regarding medications was described as too complex to understand [15]. Participants wanted more information on how to take bisphosphonates [18, 19], and in two studies, participants stated that they wanted to understand the reasons why there were specific instructions on how to take bisphosphonate medication [15, 20]. Men described wanting to know what medication was specifically suitable for them [29].

Sale et al. reported that some participants confused their bisphosphonate medication with their supplements [26]. Participants expressed a lack of guidance regarding recommended supplements [23], and the correct dose, explaining they were conflicted when choosing different sources of information to follow [26].

### Self-management

A recurrent theme across seven studies in patients with osteoporosis was the expressed view that consultations contained too much of a focus on pharmacological interventions, with advice for other non-pharmacological management, e.g. diet and exercise being relatively neglected [15, 19–22, 26, 28]. In particular, the aim of one study was to explore the level of perceived support for physical activity and supplements given by participants’ general practitioners (GPs) [21]. Many participants in this study felt their needs relating to self-management were better met by the NOS charity rather than within the DXA consultation [21]. Participants felt unsure with regard to the type and duration of activity they should be doing to help osteoporosis generally [21]. In terms of fracture recovery, patients with hip fracture described that physiotherapists were good at explaining the purpose of exercises and which exercises to do [22]. Patients valued information on mobilisation and exercise programmes [28] and particularly valued feedback on their individual progress [22]. In contrast, in a study of women’s experiences of living with vertebral fractures, participants did not feel they were given any advice on self-care, with the exception of pain relief and advice to rest, which resulted in feelings of resentment and frustration [30].

Dietary sources of calcium and vitamin D were another area of uncertainty for participants, explaining they were unsure what to eat and whether dietary sources replaced their supplements [26].

### Role of bone densitometry (DXA) and follow-up

In one study, some participants expressed confusion regarding the purpose of bone mineral density (BMD) scanning, who it was for and why only certain parts of the skeleton were measured [26]. This viewpoint was echoed in a study of outpatients from a secondary care clinic, where participants described a need for a better understanding of DXA scans and more feedback on their results [15]. In the interview study conducted by Salter et al., the lack of follow-up was described, with a participant describing the contrast between follow-up for anti-hypertensive medication consisting of blood pressure checks to ensure medication is effective, with the absence of follow-up for bisphosphonates [27]. The authors suggest that specific medication follow-up would increase confidence in medication [27], which is supported by findings in another study where participants expressed that follow-up was needed to support persistence [19].

### Factors influencing whether information needs are met

#### Perceived attitudes and knowledge of healthcare professionals

Many participants in the included studies perceived their physicians felt osteoporosis should be accepted as part of normal ageing, and that there physicians were uninterested in the condition in general [25, 30]. In addition to lack of interest, some studies reported the perception that their physicians had a poor knowledge of osteoporosis, in secondary [15] and in primary care [18]. Related to this was the perception that primary care physicians underestimated the impact of osteoporosis on their patient’s quality of life, calling for suggestions that patients need to be more involved in education programmes [21]. Where knowledge was perceived to be poor, study participants described a lack of confidence in their GP’s advice, particularly relating to physical activity [21]. In the study of patients with vertebral fractures, participants described a feeling of not being taken seriously by their doctors and a feeling that osteoporosis and vertebral fractures were of low priority [30].

#### The attitudes, beliefs and behaviours of patients

Information needs and information-seeking behaviours differed across the included studies in respect to self-efficacy, gender, age and ethnicity. McKenna et al. explored South Asian and Caucasian perceptions of support from their GP regarding non-pharmacological management [21]. The authors reported that South Asian participants differed from Caucasian participants in their information preferences in terms of tendency to consult the NOS, family members or whether they asked questions of their GP [21]. In addition, McKenna et al. consistently describe younger patients as being more proactive in seeking information, with older patients being more resigned to having osteoporosis, feeling that their actions could not change their prognosis [21]. However, in this study, the definition of ‘younger’ is not clear: the study included participants aged from 43 to 82 and quotes from two patients aged 74 and 76 are used to illustrate proactive information seeking [21].

Some studies reported that participants found it difficult to formulate questions [20]. The need for a baseline knowledge, in order to ask questions, was also described; a participant’s experience is described where she only felt confident to ask her GP questions after visiting NOS support groups [21]. The problem of lack of confidence was also acknowledged in a study of experiences of people with hip fracture, with a carer suggesting that patient advocates who can support patients in asking questions may be useful to overcome this [28].

Very little information needs were directly expressed in the studies of men [24, 29]. Male participants with osteoporosis in one study described themselves as having ‘no problems’ despite reporting daily pain or restricted activities; downplaying their symptoms or needs appeared to be part of a strategy to retain identity and a sense of masculinity [24]. Male participants in this study also described hesitation to consult their physician due to fear of receiving an osteoporosis diagnosis and how this would impact employment [24]. Efforts to consult or ask questions were sometimes to meet the information needs of female partners, rather than their own.

#### Context of information giving

Information given at one point in time, e.g. in a busy fracture clinic, may be too much to take in [26] and therefore some participants with osteoporosis (although not all) expressed preference for multiple opportunities to receive information through a series of consultations in primary care [21, 25]. Similarly, a busy ward environment was described as not conducive for information giving, or asking questions by patients with hip fracture, with the opportunity for multiple, repeated messages to be given during a stay in an intermediate care unit post fracture being valued [22]. This same study which investigated experiences of people with hip fracture emphasised the importance of involving relatives and carers in post-fracture information giving [22].

#### Types of information support

Participants wanted both written and verbal information [19, 22], and in a study where participants were asked to draw diagrams to explain their own bone health, those interviewed felt the use of more visual images would aid explanations in their routine healthcare [15]. As has been previously mentioned, ensuring that written information is understandable is key [15, 19]. Whilst many study participants already utilised other sources of information such as the NOS, participants in a Canadian study expressed a need for more community sources of information [28]. Two studies included participants that had attended support groups or group education programmes: in these studies, participants who both had and had not attended these spoke of the importance of having opportunities to meet people with similar experiences, of similar age and of similar gender [24, 25]. Others expressed an interest in being able to speak to people at a more advanced stage of osteoporosis to gain an insight into how their disease might progress, although one participant felt this might be depressing [25].

### Impact of information needs being met/unmet

#### Initiation and adherence to treatment

In two studies, the viewpoint that osteoporosis was normal for age was cited as the principle reason for non-adherence with bisphosphonates; this viewpoint (that osteoporosis was normal for age) was either reported as being directly expressed by a health professional [25] or the patient participant’s own reflection [27]. In a focus group study by Sale et al., participants reported discontinuing medication if it did not improve symptoms [26].

DXA feedback aided medication adherence by providing evidence that the medication was working [15]. Uncertainties related to side effects and harm from the medication were reported by one participant who described being anxious as to whether they were making the ‘right choice’ to take medication [17].

#### Doctor-patient relationship

Maintaining a good doctor-patient relationship is reported as being integral to the promotion of adherence with drug treatment, in a secondary care study [15]. The same study discussed how poor communication, and unmet information needs, may result in a negative perception of the doctor, and resulting break-down in doctor-patient relations [15]. The strongest narrative relating to the clinician-patient relationship was described in the study of women with vertebral fractures, although this study did not present any participant quotations, nor did it make clear who the ‘healthcare providers’ were [30]. The authors report how participants described repeated feelings that their needs were not elicited by their healthcare providers, that they felt not trusted by their doctors and that this resulted in a reluctance to then talk about their situation or seek further help [30].

#### Social and psychological consequences

In a study of patients with hip fracture, those that were considered to have unmet educational needs appeared to have an intensified fear of falling and to be ‘emotionally floundering’, ‘grasping to understand’ and ‘more likely to miscalculate risks’ [22]. In this study, tension in relationships was also described when relatives had unmet information needs. Women with vertebral fractures were described as having feelings of helplessness, underpinned by doubt and fear of an uncertain future [30]. In this study, fear of fracture and further pain influenced participant behaviour, leading to the avoidance of activities, social withdrawal and feelings of loneliness [30]. Marked anxiety and fear of fracture was also described in the study by Hansen et al., underpinned by questions not being answered by the participant’s GP [17]. The perception of osteoporosis as a female disease, reinforced by imagery on patient information leaflets, was associated with feelings of shame and embarrassment in men with the condition [24]. The authors of two included studies compare this degree of mortification to that experienced by men diagnosed with breast cancer [24, 29].

## Discussion

This review aimed to understand the information needs of patients with osteoporosis and/ or fragility fractures in order to refine research questions in this area, which is a priority for patients. The findings illustrate that one size does not fit all with a wide range of needs and preferences regarding information, as might be expected. However, the finding that core information needs prevail regarding the nature of osteoporosis, including the relationship with ageing and pain, the purpose of drug treatment, and the nature of non-pharmacological treatment, is of concern. We identified a number of barriers to information needs being met, including the perceived knowledge and attitudes of health professionals, the context in which information is given and the nature of resources supporting information exchange. Finally, we have shown that unmet information needs can have far-reaching consequences in terms of adherence to treatment, relationships with health professionals and augmenting the physical and psychosocial morbidity associated with the condition.

Wluka et al. have previously conducted an extensive review of health information needs across a range of musculoskeletal conditions [31]. This review reported that patients with rheumatoid arthritis (RA) and osteoarthritis (OA) also want to know more about the nature of the condition, as we have found. Osteoarthritis and osteoporosis are often confused [31] and both are strongly associated with ageing; however, the findings in this review and work in OA illustrate the negative impact on engagement with treatment if patients (and/or their clinicians) attribute their condition solely to ageing [31, 32]. The finding that fracture risk assessments were questioned aligns with large multicentre epidemiological study that demonstrates that postmenopausal women most at risk underestimate their own fracture risk [33]. How best to communicate fracture risk is not well established; although treatment decision aids which communicate fracture risk have been shown to improve rates of treatment adherence in small studies, they have not been qualitatively evaluated [34–36]. The review by Wluka et al. also reported that more information on self-management was wanted across all musculoskeletal conditions, with patients with OA also wanting more information on prognosis, and those with RA also needing more explanation about the purpose of medication [31]. Unique to osteoporosis it would seem is the need for more education and support around long-term treatment, to improve communication around the monitoring of the so-called silent disease and the effects of treatment. This may not be solely an issue around information as it is likely to be influenced by models of care for patients with osteoporosis, and the lack of formal procedures for monitoring the condition.

The factors we identified influencing whether information needs are met include the observation that some reported health information was too complex for some to understand, indicating low health literacy, which is likely to be a major contributor to unmet need. Health literacy is defined as the personal characteristics and social resources needed for individuals and communities to access, understand, appraise and use information and services to make decisions about health; in the UK, the majority of patient health information is too complex for 43% of the population who have limited health literacy [37, 38].

We also identified perceptions that osteoporosis was not of interest to clinicians; there is little qualitative research exploring the perceptions of primary care providers regarding osteoporosis but the limited evidence available does suggest the condition may carry a low priority when compared to other long-term conditions such as cardiovascular disease [39], and that these clinicians may have their own educational needs regarding osteoporosis [39, 40]. Furthermore, research with primary and secondary care clinicians suggest they underestimate the impact of the condition on their patients [41]. Not all information needs of patients need to be met by clinicians or specifically doctors, and many of the studies in this review describe how people use allied health professionals, e.g. pharmacists and dieticians, their social networks and other organisations to gain information. Participants expressed great satisfaction with information resources available from third sector organisations such as the NOS in previous focus groups conducted by our group [8]. However, the information giving in healthcare settings may need to be given a greater priority and be consistent with that given in other contexts.

We have described the impact of unmet information needs. We have inferred that psychosocial morbidity has occurred as a result of information needs being unmet, although in practice it is impossible to completely disentangle the impact of unmet information needs from the physical effects of the condition, e.g. vertebral fractures. However, unmet informational needs are very likely to be a determinant of health, evidenced by the association with health literacy and poor health outcomes which is well documented [42, 43]. Furthermore, and of relevance to osteoporosis, those with limited health literacy skills receive an inefficient mix of healthcare services, with care biased toward acute and emergency care rather than planned and preventative care [44, 45]. Adherence and persistence with bisphosphonates is known to be suboptimal and presents a major barrier to treating osteoporosis in a clinically and cost-effective manner. The findings of this synthesis reinforce the notion that addressing the beliefs and motivations of patients is of central importance in improving adherence [27]. In a systematic review of interventions designed to promote adherence, educational interventions targeted at patients had mixed results [46]. However, the content of these interventions and the theoretical basis on which they were framed is not well described, with only one study reporting attempts to elicit beliefs which may represent barriers to treatment [47].

### Strengths and limitations

Augmented by the help of an information specialist, this review involved a comprehensive search of available literature on expressed information needs of patients with osteoporosis/fragility fractures. The use of multiple researchers to identify relevant literature, undertake quality assessments and code data also strengthened the review. The use of very broad search terms resulted in a large number of studies to review but also meant that relevant studies were not missed. The included studies cover a wide range of different experiences, including a range of gender, age, ethnicity and nationality, thus giving a full overview of currently available evidence on information needs among patients with osteoporosis. A further strength of this review is the exclusion of studies that tested knowledge to ensure that the findings relate to patient expressed needs, unlike previous reviews in this area [31]. We used quality assessment to both inform results and underpin conclusions. However, no study was excluded based on methodological quality and the heterogeneity of studies may limit the robustness of the synthesis.

A number of other limitations are worthy of mention. First, the population of the included studies was relatively diverse including patients with or without fracture, and either with osteoporosis or deemed at high fracture risk, reflecting the change in clinical practice over the last decade to recommend fracture prevention treatments based on fracture risk rather than the presence or absence of osteoporosis. It is possible that these clinical characteristics may influence information needs. In particular, how patients make sense of being given treatment for a condition they may not have needs to be determined. One factor which limited interpretation of the findings was that it was not always clear whether authors and/or participants were referring to primary or secondary care healthcare professionals in their descriptions [30]. Issues relating to information needs may vary considerably across countries and different healthcare contexts which may limit the generalisability of our findings. While we noted that all included studies were published within the last 10 years, changes in clinical practice may make some issues more or less relevant to contemporary healthcare settings. In particular, our findings do not cover the use of the internet or the influence of the many and multiple media reports regarding osteoporosis and the safety of osteoporosis treatments that have emerged over recent years [48]. This is of particular importance because fear of side effects is an important deterrent to patients initiating treatment [49], and there is some evidence to suggest that these media stories are influencing clinicians as well as patients [50]. Finally, as only three included studies were focused on information giving and needs, and in some studies, little data was relevant for extraction, it is possible that there remain issues not covered by this review.

### Implications for practice and research

These findings raise important implications for clinical practice at the level of individual clinicians, services and wider organisations. First, health professionals (including doctors, pharmacists and nurses) involved in the care of patients with osteoporosis should reflect on to what extent they elicit or facilitate the expression of information needs and to what extent their core explanations relating to osteoporosis address issues about the nature of the condition, the purpose of medication and holistic approach to management. Second, at a service level, primary and secondary care services might consider the follow-up pathways for these patients and how these pathways are communicated to patients. Third, we suggest there is an urgent need for organisations and other providers of written information relating to osteoporosis and osteoporosis medication to ensure that material is easily understandable to those with limited health literacy. There are a number of freely available online tools that can evaluate ease of readability.

In terms of implications for research, our review has not addressed the way osteoporosis and its treatment is conceptualised in the media, and the implications of this for patient care and this remains an area where further study is needed. Understanding this societal context is critical to inform the design of public health messages. In terms of the clinician-patient encounter, the findings highlight the need to understand further healthcare professionals’ attitudes to osteoporosis, particularly that of GPs and primary care providers, and to understand the barriers to the provision of information in healthcare settings. The findings support the hypothesis that educational interventions may promote treatment adherence, but any further research in this area needs to ensure interventions are co-designed with patients, to ensure they are relevant to their needs, are applicable to a range of health literacy abilities and that the content of the interventions is explicitly mapped to the important health beliefs associated with non-adherence. Furthermore, evaluation of educational interventions should include outcomes that are patient-centred including satisfaction, self-efficacy and psychological measures. Finally, we suggest that further research is needed into the optimum ways of explaining osteoporosis and fracture risk to promote clear messages, avoid ambiguity and promote treatment persistence.

## Electronic supplementary material

ESM 1 (DOCX 17.7 kb)

### Electronic supplementary material

ESM 2 (DOCX 16.7 kb)
