# Supplementary material for: Expressed information needs of patients with osteoporosis and/or fragility fractures: a systematic review
Source: Arch Osteoporos. 2018 May 8;13(1):55. doi: 10.1007/s11657-018-0470-4 (PMC5938310; doi:10.1007/s11657-018-0470-4)
Supplement: Supplementary file 1 — (DOCX 17.7 kb) [file 11657_2018_470_MOESM1_ESM.docx]

Supplementary material 1:

Search terms

| Population | Outcomes |
| --- | --- |
| Osteoporosis  Fragility fracture | Information needs/support  Educational needs  Preferences  Information seeking  Information sources  Participant concerns  Participant satisfaction  Participant communication  Participant experience  Participant education  Participant knowledge |

**Search Strategy:**

Medline search (EBSCO 28/06/16)

| **#** | **Query** | **Results** |
| --- | --- | --- |
| S12 | S5 AND S11 | 1,739 |
| S11 | S6 OR S7 OR S8 OR S9 OR S10 | 287,640 |
| S10 | (MH "Health Education") OR (MH "Consumer Health Information+") OR (MH "Patient Education as Topic") | 131,743 |
| S9 | AB literacy OR TI literacy | 10,703 |
| S8 | AB ( patient N3 (education or communication or knowledge or concerns or information or leaflet* or preference*) ) OR TI ( patient N3 (education or communication or knowledge or concerns or information or leaflet* or preference*) ) | 55,862 |
| S7 | AB ( education N3 (support or need* or seeking or source* or resource* or health or leaflet* or preference*) ) OR TI ( education N3 (support or need* or seeking or source* or resource* or health or leaflet* or preference*) ) | 56,343 |
| S6 | AB ( information N3 (support or need* or seeking or source* or resource* or health or leaflet* or preference*) ) OR TI ( information N3 (support or need* or seeking or source* or resource* or health or leaflet* or preference*) ) | 79,241 |
| S5 | S1 OR S2 OR S3 OR S4 | 135,674 |
| S4 | AB ( fracture* N3 (osteoporo* or colles or radius or radial or femor* or hip or spinal or spine or humer* or shoulder or fragility) ) OR TI ( fracture* N3 (osteoporo* or colles or radius or radial or femor* or hip or spinal or spine or humer* or shoulder or fragility) ) | 54,918 |
| S3 | (MH "Osteoporotic Fractures") OR (MH "Radius Fractures+") OR (MH "Femoral Fractures+") OR (MH "Humeral Fractures") OR (MH "Spinal Fractures") OR (MH "Shoulder Fractures") | 61,232 |
| S2 | TI osteoporosis OR AB osteoporosis | 52,002 |
| S1 | (MH "Osteoporosis+") | 47,958 |

EMBASE search

1. osteoporosis/ or postmenopause osteoporosis/
2. osteoporosis.ti,ab,kw.
3. fragility fracture/
4. (fracture$ adj3 (osteoporo$ or colles or radial or femor$ or hip or spinal or humer$ or shoulder or fragility)).ti,ab,kw,sh.
5. 1 or 2 or 3 or 4
6. information/ or consumer health information/ or information dissemination/ or information literacy/ or information seeking/ or patient information/
7. health education/ or health literacy/ or patient education/
8. literacy.ti,ab,kw.
9. (patient$ adj3 (education or communication or knowledge or concerns or information or leaflet$ or preference$)).ti,ab,kw.
10. (education adj3 (support or need$ or seek$ or source$ or resource$ or health or leaflet$ or preference$)).ti,ab,kw.
11. (information adj3 (support or need$ or seek$ or source$ or resource$ or health or leaflet$ or preference$)).ti,ab,kw.
12. 6 or 7 or 8 or 9 or 10 or 11
13. 5 and 12
