# Supplementary material for: Expressed information needs of patients with osteoporosis and/or fragility fractures: a systematic review
Source: Arch Osteoporos. 2018 May 8;13(1):55. doi: 10.1007/s11657-018-0470-4 (PMC5938310; doi:10.1007/s11657-018-0470-4)
Supplement: Supplementary file 2 — (DOCX 16.7 kb) [file 11657_2018_470_MOESM2_ESM.docx]

**Supplementary material 2**

**Table S** Hawker et al. quality appraisal framework**^[[1]](#footnote-1)^** [12] and CASP [13] domains

| Section | Hawker et al. Appraisal Indicator | CASP Domain |
| --- | --- | --- |
| Abstract and title | Did they provide a clear description of the study? |  |
| Introduction and aims | Was there a good background and clear statement of the aims of the research? | Was there a clear statement of the aims of the research? |
| Method and data | Is the method appropriate and clearly explained? | Is a qualitative methodology appropriate?  Was the research design appropriate to address the aims of the research? |
| Sampling | Was the sampling strategy appropriate to address the aims? | Was the recruitment strategy appropriate to the aims of the research?  Was the data collected in a way that addressed the research issue? |
| Data analysis | Was the description of the data analysis sufficiently rigorous? | Was the data analysis sufficiently rigorous? |
| Ethics and bias | Have ethical issues been addressed, and what has necessary ethical approval gained?  Has the relationship between researchers and participants been adequately considered? | Have ethical issues been taken into consideration?  Has the relationship between researcher participants been adequately considered? |
| Results | Is there a clear statement of the findings? | Is there a clear statement of findings? |
| Transferability or generalizability | Are the findings of this study transferable (generalizable) to a wider population? |  |
| Implications and usefulness | How important are these findings to policy and practice? | How valuable is the research? |

1. Each item was given a score of either good (4 points), fair (3 points), poor (2 points) or very poor (1 point), giving a total score out of 36. [↑](#footnote-ref-1)
